# Reevaluating the Mutation Classification in Genetic Studies of Bradycardia Using ACMG/AMP Variant Classification Framework

**DOI:** 10.1155/2020/2415850

**Published:** 2020-02-25

**Authors:** Liting Cheng, Xiaoyan Li, Lin Zhao, Zefeng Wang, Junmeng Zhang, Zhuo Liang, Yongquan Wu

**Affiliations:** ^1^Beijing Anzhen Hospital, Capital Medical University, Beijing, China; ^2^Beijing Institute of Heart, Lung & Blood Vessel Disease, Beijing, China

## Abstract

**Purpose:**

Next-generation sequencing (NGS) has become more accessible, leading to an increasing number of genetic studies of familial bradycardia being reported. However, most of the variants lack full evaluation. The relationship between genetic factors and bradycardia should be summarized and reevaluated.

**Methods:**

We summarized genetic studies published in the PubMed database from 2008/1/1 to 2019/9/1 and used the ACMG/AMP classification framework to analyze related sequence variants.

**Results:**

We identified 88 articles, 99 sequence variants, and 34 genes after searching the PubMed database and classified ABCC9, ACTN2, CACNA1C, DES, HCN4, KCNQ1, KCNH2, LMNA, MECP2, LAMP2, NPPA, SCN5A, and TRPM4 as high-priority genes causing familial bradycardia. Most mutated genes have been reported as having multiple clinical manifestations.

**Conclusions:**

For patients with familial CCD, 13 high-priority genes are recommended for evaluation. For genetic studies, variants should be carefully evaluated using the ACMG/AMP variant classification framework before publication.

## 1. Introduction

One of the inherited bradycardias that is currently being reported is inherited progressive cardiac conduction disease (IPCCD). Progressive cardiac conduction disease (PCCD) is an unidentified, heterogeneous, life-threatening disease that manifests as progressing fibrosis of the cardiac conduction system [1]. It is characterized by a decreased conduction rate, prolonged PR interval, and widened QRS wave, and it ultimately leads to complete atrioventricular block, syncope, and even sudden cardiac death [1]. Initially, patients present with only a widened QRS wave without a bundle branch block, and later, they develop complete atrioventricular block. Abnormalities in the conduction system may be related to changes in cardiac structure and function [2]. It is currently believed that the etiology of PCCD may be related to genetic factors, valvular disease, cardiomyopathy, and autoimmune disease [3]. PCCD caused by genetic factors was originally called progressive familial heart block (PFHB) [3], and some studies directly used PCCD or IPCCD to refer to progressive conduction system diseases related to genetic factors. It is believed that PCCD is caused by the SCN5A mutation [4], and it may also be correlated with TRPM4 [5], DSP [6], and others. Genetic studies about other kinds of familial bradycardia have been published over the past decade, such as sick sinus syndrome and heart block. However, those studies have still not been summarized, and the clinical significance of the related variants is still unknown.

In 1977, Sanger et al. developed Sanger's “chain-termination” or dideoxy technique for nucleic acid sequence testing [7]. The improvement of Sanger sequencing makes DNA sequence testing for complex species available [8]. In the course of the development of next-generation sequencing (NGS), genetic testing becomes quicker, cheaper, and easier [9]. For patients who suffer from inherited cardiac disease, NGS has become a potential choice for the diagnosis, prevention, and treatment of certain diseases [9]. The relationships between inherited ion channel disease, such as long QT syndrome (LQTs) [10] and Brugada syndrome (BrS) [11], inherited cardiomyopathy, such as dilated cardiomyopathy (DCM) [12], hypertrophic cardiomyopathy (HCM) [13], and arrhythmogenic right ventricular cardiomyopathy/dysplasia (ARVC/D) [14], and variant sequencing have been well studied. However, the role of genetic sequence variants in bradycardia is still under debate.

Evaluation of sequence variants is a complex process. The integrity of both the genome and the protein being translated should be studied. In 2015, the American College of Medical Genetics and Genomics (ACMG) and the Association for Molecular Pathology (AMP) recommended an interpretative category of sequence variants and an algorithm for interpretation [15]. The ACMG/AMP classification framework is prominent in the evaluation of the Mendelian system. By evaluating the allele frequency, segregation, de novo, and protein expression, functional studies and other factors, sequencing variants can be scored as pathogenic or benign. The two parallel scoring systems divided mutations into 7 categories (Table 1). Sequence variants were then classified into a five-tier system: “pathogenic,” “likely pathogenic,” “uncertain significant,” “likely benign,” and “benign” (Table 2). By using this method, evaluated genomic variants can be quantified. With the development of evaluation methods for sequence variants, a growing number of databases have been developed. InterVar [16] is a tool implementing ACMG/AMP criteria that can automatically analyze sequence variants. LitVar [17] links genomic variants in PubMed and PMC, making functional studies achievable. With those databases, sequence variants can be evaluated properly.

At present, most of the related mutant genes reported in the literature are not analyzed according to the ACMG guidelines. In this article, we summarized and reevaluated pedigree studies of bradycardia published in PubMed from 2008/1/1 to 2019/9/1 using the ACMG/AMP variant classification framework.

## 2. Materials and Methods

### 2.1. Database Search

We searched the PubMed database by using the term “heart block” or “sick sinus syndrome” associated with “pedigree” and “‘2008/1/1'[PDAT]: ‘2019/9/1'[PDAT]” [We used the term of ((((((((((((((((((((((((Heart Block) OR Block, Heart) OR Blocks, Heart) OR Heart Blocks) OR Auriculo-Ventricular Dissociation) OR Auriculo Ventricular Dissociation) OR Auriculo-Ventricular Dissociations) OR Dissociation, Auriculo-Ventricular) OR Dissociations, Auriculo-Ventricular) OR Atrioventricular Dissociation) OR Atrioventricular Dissociations) OR Dissociation, Atrioventricular) OR Dissociations, Atrioventricular) OR A-V Dissociation) OR A V Dissociation) OR A-V Dissociations) OR Dissociation, A-V) OR Dissociations, A-V)) OR ((((((((((Hereditary bundle branch system defect) OR Heart block, progressive familial, type 1) OR Cardiac conduction defect, progressive) OR Lenegre Lev disease) OR Lenegre-Lev Disease) OR PfhbIa) OR Heart Block, Progressive Familial, Type I) OR Pfhb1a) OR Pfhbi) OR Heart block progressive, familial)) OR ((((Progressive Familial Heart Block, Type II) OR Progressive Familial Heart Block, Type Ia) OR PFHBII) OR PFHB2)) OR (((Progressive Familial Heart Block, Type Ib) OR PFHB1B) OR PFHBIB))) AND (((((((Gene) OR Cistron) OR Cistrons) OR Genetic Materials) OR Genetic Material) OR Material, Genetic) OR Materials, Genetic)) AND (“2008/01/01”[Date - Publication]: “3000”[Date - Publication])].

### 2.2. Study Selection

The aim of this study was to evaluate genetic studies of bradycardia, in addition to the inclusion criteria and exclusion criteria, as follows:

*Inclusion criterion:*

- Article published in English or have an abstract written in English
- Pedigree studies with at least one family member with bradycardia (include both sick sinus syndrome and atrioventricular block)

*Exclusion criteria:*

- Functional studies that demonstrate the main function of the sequence variants that are not focused on bradycardia
- Studies that have not demonstrated the specific mutation sites

### 2.3. Sequence Variants Analyze

#### 2.3.1. Organization of Relevant Sequence Variants

After a thorough evaluation of the related articles by two researchers, we gathered basic information about relevant sequence variants. The information included the chromosome position of the sequence variant (version: GRCh38), genomic sequence, protein sequence, dbSNP, gene, clinical manifestations, and so on.

#### 2.3.2. Clarification of Sequence Variants

The variants were named after different versions of genomics, so we used The National Center for Biotechnology Information's ClinVar database (https://www.ncbi.nlm.nih.gov/clinvar/), Online Mendelian Inheritance in Man (OMIM, https://www.omim.org), and The Human Gene Mutation Database (HGMD, http://www.hgmd.cf.ac.uk/ac/index.php) to complete detailed information on each variant.

#### 2.3.3. Use of the ACMG/AMP Classification Framework to Evaluate

According to the ACMG/AMP classification framework, we used InterVar (http://wintervar.wglab.org) (version: hg38) to evaluate sequence variants directly. With those variants that could not be defined in InterVar, we used The Genome Aggregation Database (gnomAD, https://console.cloud.google.com/storage/browser/gnomad-public/release/2.0.2/) to evaluate the allele frequency and LitVar (https://www.ncbi.nlm.nih.gov/CBBresearch/Lu/Demo/LitVar/) to evaluate whether there were relevant functional studies. Under 0.001 were defined as gnomAD. Based on information gathered in the databases and the ACMG/AMP classification framework (Tables 1 and 2), we evaluated related sequence variants and proposed a clinical judgement.

## 3. Results and Discussion

**Figure 1:**
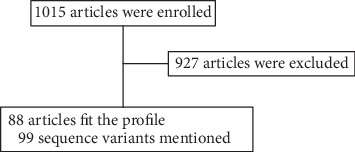
Summary of specification.

We summarized genetic studies published in the PubMed database over 11 years (Figure 1). A total 1015 articles were enrolled after searching the database. 927 articles were excluded. Finally, 88 articles fit the profile; 99 variants and 34 genes were studied in the current article.

Information in InterVar was gathered to evaluate all the sequence variants, and the relevant evidence for pathogenic and benign criteria was summarized (Table 3). For mutation cannot be defined in InterVar, we used gnomAD and ClinVar to analyze frameshift mutations (Table 4) and large fragment deletions (Table 5). We also gathered information about splicing mutations (Table 6).

We studied 88 articles, including 99 variants and 34 genes, after searching the PubMed database and identified 13 high-priority genes causing familial bradycardia, as follows: ABCC9 [18], ACTN2 [19], CACNA1C [20, 21], DES [22–27], HCN4 [28–32], KCNQ1 [33, 34], KCNH2 [35], LMNA [36, 37], MECP2 [38], LAMP2 [39], NPPA [40], SCN5A [41–45], and TRPM4 [5, 46–48] (Table 3).

We use InterVar to reevaluate APOB, CLCA2 DSG2, GJC1, GLA, GNB2, JPH2, KCNJ3, LDB3, MYBPC3, NKX2-5, NXF5, PDYN, PRKAG2, and TTN, which have been published as pathogenic variants. According to the ACMG/AMP variant classification framework, those genes should be classified into uncertain significance.

For the majority of related genes, the clinical manifestations were not unique. These mutations may lead to bradycardia, arrhythmia, myopathy, and nerve system disease. LMNA mutations may present as AVB and arrhythmia; DES, GJA5, TTN, LAMP2, and MECP2 mutations may present as AVB and myopathy; GNB5 mutation may present as CCD and nerve system disease; HCN4, KCNQ1, PRKAG2, and SCN5A mutations may present as CCD, myopathy, and arrhythmia.

Genetic diagnosis has become an inalienable part of the diagnosis, treatment, and prevention of SCD. Cardiac ion channel disease, closely related to sudden cardiac death (SCD), has been discussed for decades. In contrast, the relationship between bradycardia and genetic factors is still unclear. Syncope and SCD caused by bradycardia are life-threatening diseases. If the relationship between genetic factors and bradycardia is eliminated, SCD could be prevented.

Pedigrees of bradycardia families have been reported for decades. However, those studies are lacking. Some of the studies do not include full information about related sequence variants, and some of the studies do not list the whole family tree. In addition, the methods used to evaluate sequence variants are complex, and different centers have their own experience. It is still doubtful whether those variants are pathogenic. Therefore, ACMG/AMP promotes a guideline for thorough evaluation. By analyzing the allele frequency, segregation, de novo, protein expression, functional studies, and other factors, sequencing variants can be scored into a five-tier system: pathogenic, likely pathogenic, uncertain significant, likely benign, and benign. As accurate as the guideline may be, pathogenicity has been defined as being greater than 90% of pathogenicity [15]. According to the precise classification of pathogenicity, pedigrees of familial bradycardia can be reevaluated. InterVar [16] is a tool implementing ACMG/AMP criteria that can automatically analyze sequence variants. In this article, we used InterVar to summarize 13 high-priority genes, as follows: ABCC9, ACTN2, CACNA1C, DES, HCN4, KCNQ1, KCNH2, LMNA, MECP2, LAMP2, NPPA, SCN5A, and TRPM4 (Table 3). High-throughput sequencing (next-generation sequencing) is quite expensive. In contrast, the gene panel is cheaper and easier to analyze. We recommend that patients with a family history of bradycardia have their clinical manifestations gathered and that related pathogenic genes be highly regarded.

For future reference, multicenter studies on the epidemiology of familial bradycardia should be organized. In addition, detailed information about sequence variants should be addressed in related articles and should be evaluated under the ACMG/AMP classification framework. The relationship between bradycardia and genomic variants remains unknown, and epigenetics and modifier genes should be used to investigate the relationship between genes and diseases.

## 4. Limitation

We summarized sequence variants published in only the PubMed database. There should be more pathogenic genes studied related to bradycardia.

## 5. Conclusion and Future Direction

Only 13 pathogenic genes (99 sequence variants and 34 genes being studied) were identified after using the ACMG/AMP variant classification framework to reevaluate. For future reference, pedigree studies should be fully evaluated before being published.

For patients with familial CCD, 13 high-priority genes are recommended for evaluation. Compared to whole genome sequencing, this will increase the clinical utility of genetic testing.

## Figures and Tables

**Table 1:** Pathogenic and benign criterion based on ACMG/AMP classification framework.

Rule	Category	Rule description
Evidence of pathogenic		
Very strong	PVS1	Null variants which caused loss of function are known to be the mechanism of diseases.

Strong	PS1	Different nucleotide change caused same amino acid change with known pathogenic variants.
	PS2	De novo (confirmed maternity and paternity) in a patient with no family history and diseases.
	PS3	Functional studies supported the effect of related pathogenic variants.
	PS4	Variants' prevalence significantly increased in affected individuals than controls.

Moderate	PM1	Mutation happened in hot spot and known function domain.
	PM2	Absent (or extremely low) in large population studies.
	PM3	With recessive disease, detected in *trans* with pathogenic variants.
	PM4	Variants (in-frame deletions/insertions in a nonrepeat region or stop-loss variants) lead to changes in protein length.
	PM5	Different missense changes at known pathogenic amino acid residue.
	PM6	De novo (without confirmation of maternity and paternity).

Supporting	PP1	Variants known to be the causes affected multiple family members.
	PP2	Missense variants in a gene that have a low rate of benign missense variation are common mechanism of disease.
	PP3	Multiple lines of computational evidence support a deleterious effect on the gene or gene products.
	PP4	Phenotype specific for disease with single genetic etiology.
	PP5	Reputable source reports variants as pathogenic.

Evidence of benign		
Stand-alone	BA1	Allele frequency is >0.5% base on population database.

Strong	BS1	Allele frequency is greater than expected for disorder.
	BS2	Recessive heredity being observed in healthy adult.
	BS3	Functional studies show no pathogenic effect.
	BS4	Without segregation.

Supporting	BP1	Missense variant in gene where only loss of function is pathogenic.
	BP2	Observed in genes with overlapping function without increased disease severity or observed in *cis* with a pathogenic variant.
	BP3	Variants (in-frame deletions/insertions in a nonrepeat region or stop-loss variants) lead to changes in a repetitive region without known function.
	BP4	Multiple lines of computational evidence suggest no impact on gene or gene product.
	BP5	Variant found in a case with alternate molecular basis for disease.
	BP6	Report as benign.
	BP7	Splicing variant predict an algorithm which predict no impact to the splice consensus sequence.

**Table 2:** Sequence variant classification.

Pathogenic	1 PVS1+≥1 (PS1‐PS4)
	1 PVS1+≥2 (PM1‐PM6)
	1 PVS1 + 1 (PS1‐PS4) + 1 (PM1‐PM6)
	1 PVS1+≥2 (PP1‐PP5)
	≥2 (PS1-PS4)
	1 (PS1‐PS4)+≥3 (PM1‐PM6)
	1 (PS1‐PS4) + 2 (PM1‐PM6)+≥2 (PP1‐PP5)
	1 (PS1‐PS4) + 1 (PM1‐PM6)+≥4 (PP1‐PP5)

Likely pathogenic	1 PVS1 + 1 (PM1‐PM6)
	1 (PS1‐PS4) + 1‐2 (PM1‐PM6)
	1 (PS1‐PS4)+≥2 (PP1‐PP5)
	≥3 (PP1‐PP5)
	2 (PM1‐PM6)+≥2 (PP1‐PP5)
	1 (PM1‐PM6)+≥4 (PP1‐PP5)

Benign	1 BA1
	≥2 (BS1–BS4)

Likely benign	1 (BS1‐BS4) + 1 (BP1‐BP7)
	≥2 (BP1‐BP7)

Uncertain significant	Other criteria shown above have not met OR
	Criterion for benign and pathogenic is contradictory

OR: odds ratio.

**Table 3:** Evaluate all sequence variants using InterVar database.

Chr	Position	Ref	Alt	Gene	Criterion	Clinical manifest	Authors
12	21882785	G	A	ABCC9	Likely pathogenic	PCCD; SSS	Celestino-Soper et al. [18]
1	236731300	T	C	ACTN2	Likely pathogenic	AVB; AF	Girolami et al. [19]
2	21006288	A	T	APOB	Uncertain significance	PCCD; SSS	Celestino-Soper et al. [18]
12	2567685	G	A	CACNA1C	Likely pathogenic	SSS	Zhu et al. [20]
12	2567694	G	A	CACNA1C	Likely pathogenic	SSS	Zhu et al. [20]
12	2448997	C	T	CACNA1C	Likely pathogenic	PCCD	Gao et al. [21]
12	2504538	G	A	CACNA1C	Pathogenic	AVB; Timothy syndrome 1 (TS1)	Sepp et al. [49]
1	86447519	G	T	CLCA2	Uncertain significance	AVB; PCCD	Mao et al. [50] Tan et al. [51]
2	219425671	C	A	DES	Uncertain significance	AVB; AF	Jurcu et al. [52]
2	219418500	C	T	DES	Pathogenic	AVB	van Tintelen et al. [22]
18	31524751	A	G	DSG2	Benign/likely benign	AVB	Castellana et al. [53]
17	44805594	G	T	GJC1	Uncertain significance	AVB	Seki et al. [54]
X	101398869	A	C	GLA	Uncertain significance	HCM; AVB	Csanyi et al. [55]
7	100676751	G	T	GNB2	Uncertain significance	SSS; AVB	Stallmeyer et al. [56]
15	73329719	C	T	HCN4	Pathogenic/likely pathogenic	SSS; LVNC	Milano et al. [28]
15	73343416	A	T	HCN4	Uncertain significance	SSS; AF; LVNC	Ishikawa et al. [31]
15	73329719	C	T	HCN4	Pathogenic/likely pathogenic	SSS	Ishikawa et al. [31]
15	73323745	G	C	HCN4	Likely benign	SSS	Schweizer et al. [29]
15	73322804	C	A	HCN4	Uncertain significance	AVB	Zhou et al. [57]
20	44160305	A	T	JPH2	Uncertain significance	HCM; AVB	Vanninen et al. [58]
7	150951555	C	A	KCNH2	Pathogenic	AVB; LQT	Priest et al. [35]
2	155555534	A	C	KCNJ3	Uncertain significance	SSS; AF	Yamada et al. [59]
11	2549192	G	A	KCNQ1	Pathogenic/likely pathogenic	SSS; AF	Righi et al. [34]
X	119589315	C	T	LAMP2	Pathogenic	AVB; WPW; Danon disease	Miani et al. [39]
10	88446830	G	A	LDB3	Benign	PCCD; SSS	Celestino-Soper et al. [18]
1	156104224	C	T	LMNA	Pathogenic	AVB; VT; SCD	Glocklhofer et al. [36]
1	156104281	A	G	LMNA	Uncertain significance	AVB; HF	Petillo et al. [60]
1	156106186	G	C	LMNA	Uncertain significance	AVB; HF	Petillo et al. [60]
1	156084953	G	A	LMNA	Pathogenic	AVB; DCM	Wu et al. [61]
1	156104629	C	T	LMNA	Pathogenic	AVB; VT; SCD	Saga et al. [62]
1	156104755	T	C	LMNA	Pathogenic/likely pathogenic	AVB; muscular dystrophy; cardiomyopathy	Romeike et al. [63]
1	156084787	C	T	LMNA	Likely benign	AVB; AF	Saj et al. [37]
1	156108298	C	T	LMNA	Likely pathogenic	AVB; HCM	Francisco et al. [64]
X	153297719	G	A	MECP2	Pathogenic/likely pathogenic	SSS	Shioda et al. [38]
11	47354497	G	A	MYBPC3	Uncertain significance	AVB	Kouakam et al. [65]
5	172660006	G	A	NKX2-5	Uncertain significance	AVB; AF; DCM	Yuan et al. [66]
5	172661762	C	A	NKX2-5	Uncertain significance	AVB; congenital cardiovascular diseases (CCVD)	Pabst et al. [67]
5	172660110	G	C	NKX2-5	Uncertain significance	AVB; ASD	Xie et al. [68]
1	11907171	C	T	NPPA	Pathogenic	SSS; atrial dilatation (AD)	Disertori et al. [69]
X	101096287	G	A	NXF5	Uncertain significance	AVB; focal segmental glomerulosclerosis (FSGS)	Esposito et al. [70]
20	1961153	T	A	PDYN	Uncertain significance	PCCD	Su et al. [71]
20	1961154	C	G	PDYN	Uncertain significance	PCCD	Su et al. [71]
7	151560613	A	G	PRKAG2	Uncertain significance	HCM; AVB	Thevenon et al. [72]
3	38550326	G	T	SCN5A	Uncertain significance	SSS	Chen et al. [73]
3	38603929	G	C	SCN5A	Uncertain significance	AVB	Nikulina et al. [74]
3	38556532	T	C	SCN5A	Uncertain significance	SSS	Hothi et al. [41] Asadi et al. [75]
3	38550734	A	C	SCN5A	Uncertain significance	SSS	Abe et al. [76]
3	38613790	C	T	SCN5A	Likely pathogenic	SSS	Abe et al. [76]
3	38566426	C	T	SCN5A	Pathogenic	AVB; DCM	Watanabe et al. [77]
3	38550899	T	A	SCN5A	Uncertain significance	SSS	Ishikawa et al. [31]
3	38581137	G	A	SCN5A	Likely benign	AVB	Hu et al. [78]
3	38581002	C	T	SCN5A	Uncertain significance	SSS; AFL; AF	Moreau et al. [79]
3	38633207	G	T	SCN5A	Uncertain significance	AVB	Thongnak et al. [80]
3	38613787	G	A	SCN5A	Uncertain significance	PCCD; SSS	Baskar et al. [81] Celestino-Soper et al. [18]
3	38597787	C	A	SCN5A	Likely pathogenic	SSS; AFL	Selly et al. [82]
3	38630342	T	A	SCN5A	Pathogenic/likely pathogenic	SSS; AFL; VT	Holst et al. [43]
3	38575424	C	A	SCN5A	Uncertain significance	AVB; DCM	Ge et al. [83]
3	38551477	A	T	SCN5A	Likely pathogenic	SSS; AVB	Robyns et al. [84]
3	38560398	G	A	SCN5A	Pathogenic	AVB	Thongnak et al. [80]
3	38550968	C	A	SCN5A	Uncertain significance	SSS	Abe et al. [76]
19	49196760	G	A	TRPM4	Uncertain significance	PCCD	Liu et al. [47]
19	49157885	G	A	TRPM4	Pathogenic	PCCD; SSS	Kruse et al. [48]
19	49167950	G	A	TRPM4	Benign	AVB; VT	Bianchi et al. [46]
19	49196790	A	G	TRPM4	Likely benign	PCCD	Daumy et al. [5]
19	49202140	A	T	TRPM4	Uncertain significance	AVB; VT	Bianchi et al. [46]
19	49171597	A	G	TRPM4	Uncertain significance	AVB	Stallmeyer et al. [85]
19	49200395	A	G	TRPM4	Pathogenic	AVB	Stallmeyer et al. [85]
19	49168301	C	T	TRPM4	Pathogenic	PCCD	Liu et al. [47]
19	49182608	G	A	TRPM4	Uncertain significance	AVB	Syam et al. [86]
19	49188641	G	A	TRPM4	Uncertain significance	AVB	Syam et al. [86]
19	49183108	C	T	TRPM4	Uncertain significance	PCCD	Liu et al. [47]
19	49196597	T	C	TRPM4	Uncertain significance	AVB	Stallmeyer et al. [85]
2	178569522	G	T	TTN	Uncertain significance	SSS	Zhu et al. [20]

**Table 4:** Using ClinVar to analysis frameshift mutation.

Genome AD	Chr	dbSNP	Gene	Variant	Functional study	Criterion
—	—	—	ALG13	c.383+2821_383+2822delinsTT	—	—
—	Chr2:219418955-219418982	rs1114167332	DES	c.493_520del28insGCGT	—	Pathogenic
—	—	—	DSC2	c.2688_2688delinsGAA	—	—
—	—	—	EXT2	c.1101_1102delAG (E368Kfs^∗^18)	—	—
—	Chr1:156130627-156130629	rs794728597	LMNA	c.367_369delAAG	Pathogenic	Likely pathogenic
—	—	—	LMNA	c.364_366AAG	—	—
—	—	—	LMNA	c.103-105del CTG	—	—
			LMNA	815_818delinsCCAGAC		
—	—	—	MYL4	c.234delC	—	—
—	Chr5:173232761	rs587784067	NKX2.5	c.959delC	—	Conflicting interpretations of pathogenicity
—	—	—	SCN5A	c.2401_2409delinsTCC	—	Uncertain significant
—	—	—	SCN5A	c.5355_5354delCT	—	Uncertain significant
—	—	—	SCN5A	c.5368 GNA	—	—
—	—	—	SCN5A	c.3142_3153de-l12ins11	—	—
			MYH6	delE933		
			MYL4	c.234delC		

**Table 5:** Using InterVal to analysis large fragment deletion.

Genome AD	Chr	dbSNP	Gene	Variant	Functional study
—	—	—	DES	Deletion-insertion mutation (c.1045-1063 del/G ins), deleting 7 amino acids (Met349-Arg355) and inserting 1 amino acid (Gly349)	—

**Table 6:** Analyzing splicing mutation.

Genome AD	Chr	dbSNP	Gene	Variant	Functional study
—	—	—	HCN4	c.1737+1G>T	—
—	Chr:1:156130615	—	LMNA	c.357-2A>G	—
—	—	—	LMNA	c.357-1G>T	—
—	—	—	LMNA	IVS9-3C>G	—
G = 0.00001	Chr3:38562413	rs397514447	SCN5A	c.3963+2T>C	—
—	—	—	SCN5A	c.1141-2A>G	—
—	—	—	SCN5A	c.-225-820T>C	—
—	—	—	TGF beta 1	c.4246-2A>G	—
—	—	—	MYH6	c.2292+2T>C	—

## Data Availability

There are no restrictions on data access of this paper. All works have been provided in this paper
